# Binary classification of protein molecules into intrinsically disordered and ordered segments

**DOI:** 10.1186/1472-6807-11-29

**Published:** 2011-06-22

**Authors:** Satoshi Fukuchi, Kazuo Hosoda, Keiichi Homma, Takashi Gojobori, Ken Nishikawa

**Affiliations:** 1 Center for Information Biology & DNA Data Bank of Japan, National Institute of Genetics, Yata 1111, Mishima, Shizuoka 411-8540, Japan; 2 Department of Bioinformatics, Maebashi Institute of Technology, Kamisadori 460-1, Maebashi, Gunma 371-0816, Japan

## Abstract

**Background:**

Although structural domains in proteins (SDs) are important, half of the regions in the human proteome are currently left with no SD assignments. These unassigned regions consist not only of novel SDs, but also of intrinsically disordered (ID) regions since proteins, especially those in eukaryotes, generally contain a significant fraction of ID regions. As ID regions can be inferred from amino acid sequences, a method that combines SD and ID region assignments can determine the fractions of SDs and ID regions in any proteome.

**Results:**

In contrast to other available ID prediction programs that merely identify likely ID regions, the DICHOT system we previously developed classifies the entire protein sequence into SDs and ID regions. Application of DICHOT to the human proteome revealed that residue-wise ID regions constitute 35%, SDs with similarity to PDB structures comprise 52%, while SDs with no similarity to PDB structures account for the remaining 13%. The last group consists of novel structural domains, termed cryptic domains, which serve as good targets of structural genomics. The DICHOT method applied to the proteomes of other model organisms indicated that eukaryotes generally have high ID contents, while prokaryotes do not. In human proteins, ID contents differ among subcellular localizations: nuclear proteins had the highest residue-wise ID fraction (47%), while mitochondrial proteins exhibited the lowest (13%). Phosphorylation and *O*-linked glycosylation sites were found to be located preferentially in ID regions. As *O*-linked glycans are attached to residues in the extracellular regions of proteins, the modification is likely to protect the ID regions from proteolytic cleavage in the extracellular environment. Alternative splicing events tend to occur more frequently in ID regions. We interpret this as evidence that natural selection is operating at the protein level in alternative splicing.

**Conclusions:**

We classified entire regions of proteins into the two categories, SDs and ID regions and thereby obtained various kinds of complete genome-wide statistics. The results of the present study are important basic information for understanding protein structural architectures and have been made publicly available at http://spock.genes.nig.ac.jp/~genome/DICHOT.

## Background

Understanding of human proteins is doubtlessly essential for both basic and applied sciences. With protein structures accumulating and protein structure prediction improving, it is becoming increasingly accurate to assign structural domains (SDs) to amino acid sequences. With the advent of the genome era, protein structural annotations became one of the most important kinds of information on genome sequences [1-3], because SDs are structural as well as functional units of proteins. Two sequences with sequence similarity can have similar 3D structure. So-called profile methods, such as PSI-BLAST [4] and profile hidden Markov model [5], enable us to infer protein 3D structure from amino acid sequences by searching for subtle homologies that are difficult to detect with usual homology search programs such as BLAST. Even though structural genomics projects have expanded structural information, no less than 50% of regions of human proteins remain without structural annotations [1].

The discovery of intrinsically disordered proteins (IDPs) has brought a paradigm change to structural biology [6-8]. IDPs are those that do not assume any stable 3D structure by themselves under physiological conditions. Some proteins are fully composed of intrinsically disordered (ID) regions while others contain long ID regions. Indeed, state-of-the-art experiments at the single-molecular level using high-speed atomic force microscopy [9] showed that ID regions are in flexible and extended conformation in their free state. IDPs are involved in crucial biological processes such as signal transduction, transcription control [10-13]. Typically functional IDPs switch to more ordered states or fold into stable secondary or tertiary structures upon binding to targets, a phenomenon known as coupled folding and binding [14-20]. Interestingly it was found that phosphorylation sites preferentially reside in ID regions [21].

Protein sequences in ID regions have characteristic amino acid compositions, which can be used for prediction of ID regions [22-27]. A genome-wide prediction of ID regions revealed that the residue-wise ID fractions in archaea, bacteria and eukaryotes are 4%, 6%, 19%, respectively [27]. Eukaryotic transcription factors are salient examples of IDPs: the average ID fraction of human transcription factors was estimated to be as high as 49% [28]. However, the conventional prediction methods only identify possible ID regions, without assigning the remainder as SDs. In order to remedy this defect, we developed the DICHOT method [29] which divides the entire amino acid sequence of a query protein into SDs and ID regions.

In addition to conventional methods of SD assignment [1] and ID prediction, the DICHOT system introduces sequence conservation as a third factor, based on the observation that ID regions are less conserved than structural regions are [29]. Consequently SDs assigned by DICHOT include not only SDs of known structure (KDs), i.e., those with sequence similarity to existing PDB entries, but also novel SDs, i.e., those without similarity to PDB entries. The novel SDs, termed cryptic domains (CDs) in this study, are globular structures whose 3D structures have not been determined. Here we apply DICHOT to the human proteome to estimate the fractions of protein residues in KDs, CDs, and ID regions, and then compare them with those of other model organisms.

## Results

### Application of DICHOT to human proteins

The DICHOT system classifies the entire region of an amino acid sequence into SDs and ID regions. First, potential SDs are detected by using the sensitive homology-search tools such as PSI-BLAST and HMM, and are masked. Then, the remaining regions are further classified into the two categories by a disorder prediction program, DISOPRED2, and CLADIST, which is a newly developed disorder prediction program described previously [29]. DISOPRED2 is a method that employs SVM trained by PSSM obtained by PSI-BLAST. It is trained by SDs and missing residues in the PDB. Only 3% of total residues in KDs overlapped with ID regions predicted by DISOPRED2. This result suggests that DISOPRED2 mainly identifies ID regions outside of KDs.

A benchmark test revealed that the error rate of the system is less than 3% [29]. As the DICHOT system was optimized for transcription factors, we added rules in the present study in order to deal with human proteins other than transcription factors. We made additional rules for transmembrane regions, signal sequences for secretion, and fibrous sequences such as collagen so that all of these are classified as SDs of known structure (see Materials and Methods). Binary classification of proteins into SDs and ID regions inevitably produces CDs because not all of the SDs has experimentally determined 3D structures. The uniqueness of DICHOT rests on its ability to identify CDs, as conventional disorder-prediction methods do not identify cryptic domains.

**Figure 1:**
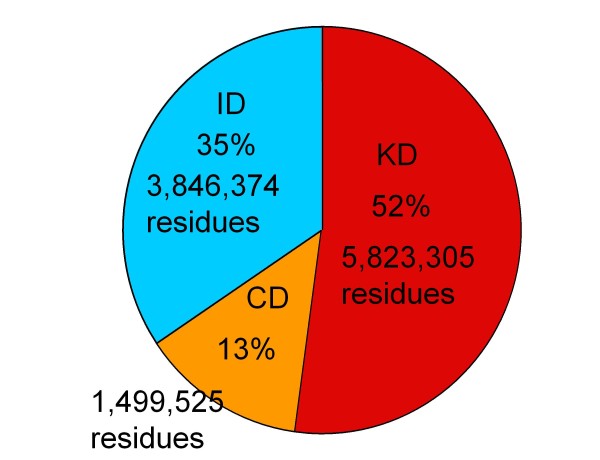
**Fractions of KDs, CDs, and ID regions in the human proteome**. The DICHOT system was applied to all human proteins in the Swiss-Prot database. The red, orange and blue regions represent SDs of known structure (KDs), cryptic SDs (CDs), and ID regions, respectively. The fraction and the number of total residues in each section are shown.

DICHOT has been applied to 20,333 human proteins taken from the Swiss-Prot database [30] containing a total of 11,169,204 residues. 35% of the residues were judged to be in ID regions, 52% fell in KDs, while13% were predicted to be in CDs (Figure 1). The ID fraction in the present study is higher than that (22%) reported [27]. This makes sense as the DICHOT system classifies previously ambiguous sections into ID and SD segments and thereby increases both fractions. Therefore, the above-mentioned fractions are the first precise structural evaluation of the human proteome. The detailed results on individual human proteins can be accessed at http://spock.genes.nig.ac.jp/~genome/DICHOT. The on-demand system is also included in the FUJI database (http://fujidb.genes.nig.ac.jp/fujidb/input_form.php?lang=en).

### Application of DICHOT to proteins of other organisms

**Figure 2:**
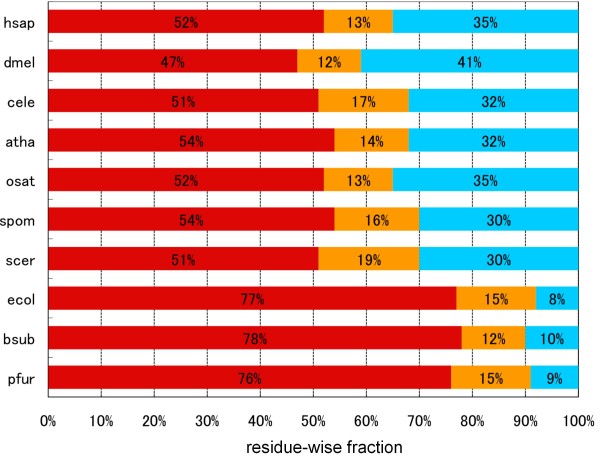
**Fractions of KDs, CDs, and ID regions in 10 model organisms determined by the DICHOT system**. The species names are abbreviated in four letters: hsap, dmel, cele, atha, osat, spom, scer, ecol, bsub, and pfur stand for *Homo sapiens*, *Drosophila melanogaster*, *Caenorhabditis elegans*, *Arabidopsis thaliana*, *Oryza sativa*, *Schizosaccharomyces pombe*, *Saccharomyces cerevisiae*, *Escherichia coli*, *Bacillus subtilis*, and *Pyrococcus furiosus*, respectively. KDs, CDs, and ID regions are color-coded as in Figure 1. The actual fraction of each section is also shown.

We applied the DICHOT system to the proteomes of several model organisms and compared the results with that of the human proteome (Figure 2). A clear difference in the pattern of SD and ID fractions between eukaryotes and prokaryotes is seen: for instance, the ID fraction ranges from 30% (*Saccharomyces cerevisiae *and *Schizosaccharomyces pombe*) to 41% (*Drosophila melanogaster*) in eukaryotes, whereas it is equal to or less than 10% in bacteria (*Escherichia coli *and *Bacillus subtilis*) and archea (*Pyrococcus furiosus*). In agreement with the previous report [27,31,32], ID regions are more frequent in eukaryotes than in prokaryotes and the proteome of *D. melanogaster *has a higher ID fraction than other eukaryotes.

**Figure 3:**
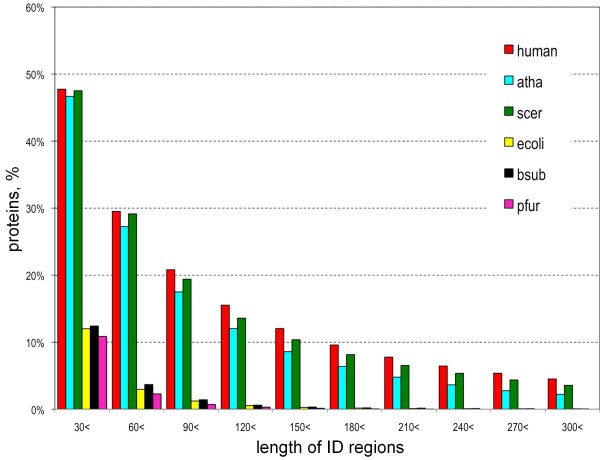
**Percentage of IDPs with contiguous ID regions longer than the specified length**. Bars in different colors represent percentages of proteins in respective species with consecutive residues more than the indicated number assigned as ID regions. See the legend to Figure 2 for the shortened names of the six species.

The eukaryote-prokaryote difference in ID fraction becomes more striking if we limit our attention to long ID regions. When the percentage of IDPs is plotted against contiguous ID regions of lengths larger than a certain residues, the difference becomes more marked for greater length cut-offs (Figure 3). The ratio of the human and *E. coli *ID fractions is about one fourth at length in excess of 30 amino acids (leftmost bars), but rapidly decreases as the cutoff length of contiguous ID regions increases, and the ID fraction in *E. coli *is negligible for lengths longer than 150. Because eukaryotic proteins are generally longer than prokaryotic ones, it is possible that the above-mentioned propensity of long ID regions is a consequence of the dependence on protein lengths. To test this possibility, we took the same statistics using datasets of both human and *E. coli *proteins whose lengths are limited to a certain range (200 – 400 residues), because the average and the standard deviation of *E. coli *proteins are approximately 300 and 200, respectively. As the resultant distributions (see Additional file 1) turned out to be mostly identical to those presented as Figure 3, significant length dependency does not exist.

In contrast to the ID fraction, it is interesting to see that the fractions of CDs do not vary greatly among organisms: there is no clear eukaryote-prokaryote divide with the highest and the lowest fractions being 19% (*S. cerevisiae*) and 12% (*D.melanogaster *and *B. subtilis*), respectively (orange bars in Figure 2).

### Dependence of ID fraction on subcellular localization

**Figure 4:**
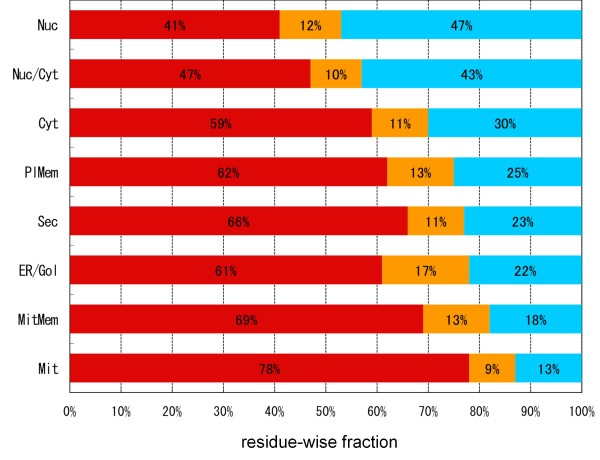
**Fractions of KDs, CDs, and ID regions in different subcellular localizations**. The human proteins were classified according to subcellular localizations and the fractions of KDs, CDs, and ID regions in each of the localizations were calculated. Nuc: nuclear, Nuc/Cyt: nuclear and cytoplasmic, Cyt: cytoplasmic, PlMem: plasma membrane, Sec: secreted, ER/Gol: ER and Golgi, MitMem: mitochondorial membrane, and Mit: mitochondorial proteins. The bars are colored as in Figure 1.

It was Ward et al. [27] who first found an uneven subcellular distribution of the fraction of ID regions through analysis of yeast proteins after classifying them according to the GO (gene ontology) categories of subcellular localization. Using DICHOT, we performed a similar analysis on human proteins. We employed the Swiss-Prot annotation for classification of individual proteins into different subcellular localizations, such as the nucleus, the cytoplasm, and the plasma membrane. As multiple subcellular localizations are occasionally assigned to single proteins in Swiss-Prot, we excluded them from the analysis except for those whose subcellular localizations are annotated as "nucleus and cytoplasm". (See Additional file 2 for the number of proteins assigned to each subcellular localization.) We determined the average KD, CD, and ID fraction in each subcellular localization and presented them in Figure 4, where the localizations were arranged in the descending order of ID fraction. Nuclear proteins contain a conspicuously high fraction (47% on average) of ID regions. Considering that transcription factors are localized to the nucleus, the particularly high prevalence of ID regions in nuclear proteins is at least partially attributable to a high ID fraction (62%) of transcription factors [29]. Conversely, mitochondrial proteins contain the lowest fraction of ID regions (13%). This is consistent with the fact that ID fractions of prokaryotic proteins are generally low (Figure 2), as mitochondria are generally thought to have originated from engulfed bacteria as endosymbionts. This point is further discussed below.

Selection of ID-rich proteins first and subsequent extraction of the corresponding Swiss-Prot keywords showed over-representation of some keywords, including "transcription", "transcription factors", "DNA binding", "RNA binding", and "mRNA processing" [27,33,34]. These keywords are strongly associated with nuclear proteins, but not with proteins of other subcellular localizations, for instance, extracellular secreted proteins. Classification of proteins into intracellular (in the cytoplasm and the nucleus) and extracellular (secreted, the ER and the Golgi apparatus) types reveals that ID regions exist more in intracellular than in extracellular proteins (Figure 4). As membrane proteins have both intracellular and extracellular domains, it makes sense that they have an intermediate frequency of ID regions. In fact, when membrane proteins were divided into intracellular and extracellular domains, the former domains had more ID regions than the latter [35]. Taken together, human IDPs preferentially populate the intracellular environment than the extracellular milieu.

### Prevalence of various protein features in the ID region

**Figure 5:**
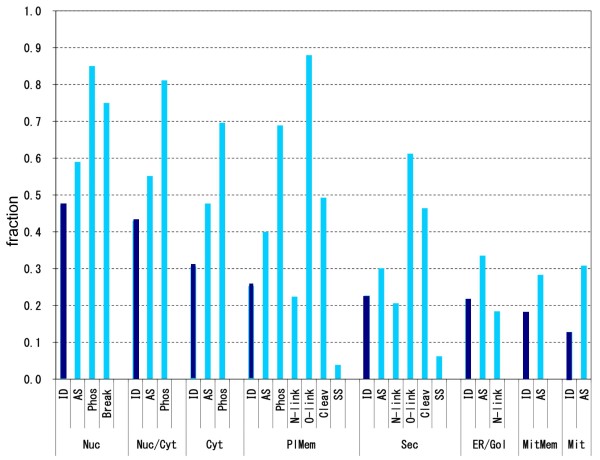
**Frequencies of various modifications occurring in ID regions**. The protein modifications considered are: alternative splicing (AS) boundary, phosphorylation (Phos), breakpoint in chromosomal translocation (Break), *N*-linked and *O*-linked glycosylation (N-link, *O-*link), cleavage by protease (Cleav), and disulfide bond (SS). The subcellular localizations are abbreviated as in Figure 4. The bar of phosphorylation in nucleus, for instance, indicates the fraction of phosphorylation sites occurring in ID regions in nuclear proteins. If phosphorylation sites were evenly distributed throughout SDs and ID regions in nuclear proteins, the fraction would be the same as the corresponding fraction of ID regions. The internal standards are presented as navy blue bars.

The Swiss-Prot database has annotations on sites of post-translational modifications such as phosphorylation, *N*-linked and *O*-linked glycosylation, sites of cleavage for hormone production, and breakpoints for fusion protein formation in translocation as well as sites of alternative splicing (AS). Each site was judged to be either in an SD or ID region, without distinguishing CDs from KDs. Then the average fractions of modifications in ID regions were determined in each subcellular localization. All the results are shown in Figure 5, where each column presents the fraction of the indicated modifications or AS sites occurring in ID regions. In each subcellular localization, the leftmost column labeled "ID" corresponds to the ID fraction in Figure 4, serving as the internal standard.

The frequency of AS occurrence is always higher than the respective inner standard, regardless of subcellular localizations. This indicates that AS occurs preferentially in ID regions [36], whose significance is considered in Discussion. Phosphorylation sites are predominantly found in proteins in the intracellular localizations, i.e. the nucleus, the cytoplasm, and presumably the intracellular domains of membrane proteins, and are more frequently found in ID regions than in SDs (Figure 5) as previously reported [21]. By contrast, *N*-linked and *O-*GalNAc glycosylation sites are found nearly exclusively in extracellular proteins, that is, secreted proteins, those in the ER and Golgi as well as the extracellular domains of membrane proteins. As already reported [37], *O*-GalNAc modification significantly prefers IDs to SDs, whereas *N*-linked glycosylation shows no such preference (Figure 5). Loss of *O*-GalNAc modification in human low density lipoprotein receptor, for instance, makes the receptor vulnerable to proteolytic cleavage [38] and elimination of an *O*-GalNAc modification site in human transferrin receptor renders the region susceptible to cleavage [39]. It makes structural sense that the breakpoints in translocation and cleavage sites for peptide-hormone production tend to be in ID regions. As is well known, disulfide bond formation is limited to extracellular proteins (secreted and membrane proteins), serving to stabilize them in extracellular environment. Figure 5 indicates that disulfide bond formation occurs mostly in SDs, and rarely in ID regions of proteins.

## Discussion

Using the DICHOT system, the present study has revealed, for the first time, the concrete ratio of intrinsically ordered and disordered segments in the human proteome: on the average 65% of human protein residues are in SDs, while the remaining 35% fall within ID regions. Since SDs so identified include those with unknown structures (CDs), the application of DICHOT unprecedentedly uncovers the locations of CDs along the sequence and produces to the fractions of CDs in the human proteome (Figure 1) and others (Figure 2). The fact that DICHOT can locate not only KDs and ID regions, but also CDs is a novel feature of DICHOT not found in conventional disorder-prediction methods. As structural genomics progresses, the fraction of CDs is expected to decrease. However, the ratio of ordered/disordered segments will not be affected and will remain unchanged.

Concerning the accuracy of the DICHOT assignment, the fraction of KDs is determined most accurately. In our definition, KD contains globular domains (homologous to PDB structures), trans-membrane regions, coiled-coil regions, fiber proteins, and signal sequence. The fraction of KDs is the sum of the fractions of these regions, each of which can be accurately determined. This fraction is 52% (Figure 1), and the confidence of this value is very high. On the other hand, the CD fraction is expected to be the least accurate. However, the human proteome doubtlessly contains some proteins with unknown structural domains. The upper limit of ID is 48% if proteins are assumed to consist of KDs and IDs only, but the limit is lowered if we consider the existence of CDs. The present result shows 35% and 13% for the fraction of IDs and CDs, respectively, although admittedly the figures may contain some errors caused by misassignments. Although we will improve the classification as needed, we can say with confidence that the fraction of ID cannot exceed 48%.

The present study has revealed not only the ID fraction but also the fraction of SDs in proteins. Individual KDs were located and identified by homology searches against the PDB and SCOP databases. The total number of folds (defined as SCOP superfamilies [40]) identified in the human proteome was 943, which should be compared with the corresponding number in *E. coli*, 690. The present study revealed the fraction of CDs as well. We expect CDs to contain many new folds and consider it interesting to estimate the number of new folds that will be experimentally determined in future [41]. However, clustering of sequences in CD and identifying folding units are beyond the scope of the present study.

Then, we approached this problem from another angle, using Pfam domains. Pfam defines protein domains based on sequence conservation [42]. Because Pfam uses only sequence information, some Pfam domains cover not only KDs and CDs, but also ID regions in some cases [43]. Such occasional overlaps of Pfam domains and ID regions prevented us from using Pfam in the DICHOT system to assign SDs and IDs. Because DICHOT does not utilize Pfam, Pfam-based analyses can serve as an independent check on DICHOT. We estimated the fractions covered by Pfam domains in KDs, CDs, and ID regions using HMM Pfam search, and tabulated the results (Table 1). The Pfam fractions in KDs, CDs, and ID regions are 60.6%, 26.6%, and 8.5%, respectively. The reason why the coverage of KD is less than 100% may be that Pfam domains do not completely correspond to PDB structures, and Pfam domains do not contain trans-membrane regions and signal sequences, both of which are regarded as KDs in the present study. That 8.5% of ID regions is covered by Pfam domains does not imply the existence of structural domains in IDs, but that 8.5% of Pfam domains are in ID regions [44,45]. The significantly higher Pfam coverage of CDs than that of ID regions suggests that these two indeed differ. Although CDs and KDs in the present study are expected to be similar, the Pfam coverage of CDs is significantly lower than that of KDs. The discrepancy may be attributable to the preferential assignment of Pfam domains to well-studied regions, e.g. structurally determined regions (KDs), in contrast to the assignment of all probable structural domains (CDs and KDs) without human bias by DICHOT.

**Table 1:** Estimation of number of folds in the CD regions.

	KDs	CDs	ID regions
#total (residues)	5,823,305	1,499,525	3,846,374
#Pfam hit (residues)	3,529,535	398,328	326,800

Pfam coverage	60.6%	26.6%	8.5%

#unique Pfam domains	2450	1348	1851
#SCOP superfamilies	943	519^(1)^	-

Residue-wise fraction (Fig. 1)	52%	13%	35%
#SCOP superfamiles	943	236^(2)^	-

(1) is calculated by  and (2) is calculated by ,

where #unique_Pfam_CD is the number of unique Pfam families in CDs, #unique_Pfam_KD is the number of unique Pfam families in KDs, #residue_CD is the number of residues in CDs, #residue__KD is the number of residues in KDs, #SCOP_fold_KD is the number of SCOP folds (superfamilies) in KDs.

The numbers of unique Pfam domain in KDs, CDs, and ID regions are 2,450, 1,348, and 1,851, respectively (Table 1). On the other hand, the number of SCOP superfamilies found in KDs is 943 as already mentioned. Assuming the constancy in the ratio of the number of SCOP superfamilies and the unique Pfam domains in KDs and CDs, we estimated the number of SCOP superfamilies in CDs as 519 (see also Table 1). This figure is considerably larger than the one (236 in Table 1) estimated under a simple assumption that the number of SCOP superfamilies per residue is constant in KDs and CDs. This discrepancy may be explained by the following notion: KDs contain many ubiquitous folds found frequently in different proteins and cover a large fraction of regions. The 3D structures of these folds tend to be preferentially determined because their prevalence attracts the interest of many researchers. By contrast, the structures in CDs are likely to contain rarer folds. More fold classes of rarer folds are needed to cover the same regions as ubiquitous fold classes do. We will be able to make a more precise inference of the number of fold classes as the fraction of CDs decreases and consequently improves the precision of the number estimate of new folds [46,47].

Another notable result of the present study is the sharp distinction of ID regions in eukaryotes and prokaryotes, consistent with the previous observation by Ward et al. [27]. A clear difference was detected not only in the residue-wise ID fraction (Figure 2), but also in the protein-wise percentage of IDPs (Figure 3). These observations suggest that IDPs in eukaryotes and prokaryotes differ not merely quantitatively but qualitatively: almost all longer ID regions seem to associate with eukaryotes (Figure 3), while shorter ID regions in prokaryotes seem to come from linkers between domains and N- and C- terminal regions. In this context, mitochondrion is an intriguing case because it is a cellular organelle of the eukaryotic cell on the one hand, while it is generally agreed to be a descendant of bacteria on the other. At first glance, the low ID fraction of mitochondrial proteins (Figure 4) appears consistent with the general scarcity of IDs in prokaryotic proteins (Figure 2). However, a closer inspection reveals that the former value (13%) is larger than that of *E. coli *(8%). The difference between mitochondria and *E. coli *becomes more apparent when the length distributions of ID regions are compared: the fraction of ID length over 30 in *E. coli *is 12% (Figure 3), while the corresponding fraction of mitochondria is more than 30% (see Additional file 3). This inconsistency may come from the fact that mitochondria not only have proteins of prokaryotic origin, but also have those of eukaryotic provenance incorporated after endosymbiosis [48,49]. In fact, we can identify a number of mitochondrial proteins that have homologs in eukaryotes but not in prokaryotes. To name a few, CPT2_HUMAN (Swiss-Prot Accession Number: P23786), DBLOH_HUMAN (Q9NR28), KCRS_HUMAN (P17540), LETM1_HUMAN (O95202), MPCP_HUMAN (Q00325), PTH2_HUMAN (Q9Y3E5), STAR_HUMAN (P49675), and TFAM_HUMAN (Q00059) fit this category. The results shown in Figure S2 (see Additional file 3) suggest that mitochondrial proteins consist of two distinct types: one with lower ID fractions (prokaryotic type) and the other with higher ID fractions (eukaryotic type). More detailed analyses are required to give a definitive conclusion on this subject.

Protein functions and structures are influenced by subcellular localizations [50]. The present study revealed that the ID fractions of proteins also depend on their subcellular localizations. Although Ward et al. [27] reported the dependence of ID fraction on different subcellular localization categories, the complete division of protein molecules into SDs and ID regions by DICHOT made it possible to clarify quantitative diversity among subcellular localizations. The present results agree with the previous observation [27] that ID regions are most abundant in nuclear proteins, while they are most scarce in mitochondrial proteins (Figure 4). Binary classification into SDs and ID regions in individual proteins made it easy to examine which modifications occur more frequently in SDs or ID regions. Figure 5 shows that phosphorylation and *O*-linked glycosylation occur more frequently in ID regions than in SDs, while disulfide bonds exhibit the opposite tendency. Considering that *O*-linked glycosylation is added to residues in the extracellular domains of proteins and that unmodified ID regions are vulnerable to proteolytic cleavage, *O*-linked glycans probably serve as a protective measure within the ID regions of proteins in the extracellular environment. Complementarily, disulfide bonds stabilize SDs of extracellular proteins and presumably guard them against proteolysis.

Figure 5 also demonstrates that AS preferentially occurs in ID regions, confirming the previous report [36]. Intriguingly the preferential occurrence of AS in ID regions is observed regardless of subcellular localizations: in all the localizations, the AS bar is higher than the internal standard (Figure 5). Interpretation of this preference is not trivial. To understand the phenomenon of AS, we must consider its effects on mRNA and protein separately. Although AS causes no physiological problems in the mRNA produced, it may have serious detrimental effects on the encoded protein: if changes in amino acid sequence such as the deletion of the entire sequence encoded by an exon occur within structural domains, the proteins would no longer be able to maintain the original 3D structures [51,52]. This difficulty can be dissolved by IDPs consisting not only of SDs but also of ID regions: if AS events occur within ID regions, the changes in sequence will not affect the protein structures [7,8,36]. From the evolutionary viewpoint, the biasing of AS towards IDs implies natural selection operating at the protein level. In general, AS is directly associated with transcription. If there was no feedback from the protein level, AS events would occur in direct proportion to exon boundaries, as hypothesized by Moult et al. [53,54]. We plotted exon boundaries at either ID regions or SDs as in Figure 5, and obtained results presented in Figure S3 (see Additional file 4). The fraction of exon boundaries occurring in ID regions is much lower than the corresponding fraction of AS boundaries in any of the localization categories. Thus, the data do not support the splicing-noise model of AS [53,54] which states that most AS events are a consequence of stochastic noise and of no functional significance. Our results instead suggest that natural selection is in fact operating on AS events at the protein level.

## Conclusions

We developed the system, DICHOT, for classifying structured/unstructured regions of proteins. The application of DICHOT to the proteomes can bring a basis for understanding protein domain architectures. In particular, the complete classification into SD/ID regions is fruitful for planning experiments, and CDs are intriguing targets for structural biology. The results can be accessed at http://spock.genes.nig.ac.jp/~genome/DICHOT.

## Methods

The details of the DICHOT system were described in our previous report [29]. Briefly, the system consists of two sections, a SD assignment and a classification of the remaining regions. The first section assigns SDs by BLAST, reverse PSI-PLAST, and HMMer, and the second section divides the remaining sections into SDs and ID regions by a combination of DISOPRED2 and a newly developed ID/SD classification program called CLADIST that makes use of amino acid composition and sequence conservation. All ID regions predicted by both DIOPRED2 and CLADIST are accepted. A region predicted to be ID by DISOPRED2, but assigned as SD by CLADIST is regarded as ID. Regions predicted as SD by CLADIST and unassigned by DISOPRED2, are judged as CDs if they are longer than 30 amino acid residues. We note that functional ID regions are occasionally found in the PDB because their structures bound to their partners have been determined. To avoid these regions to be erroneously classified as SDs, regions aligned with short PDB sequences (less than 50 amino acids) are left unclassified. These regions are judged in the second section, where the regions not assigned as SDs are classified by disorder prediction.

Because the system was developed using typical IDPs, human transcription factors [29], we modified the system before applying it to the whole proteomes: the modified system uses the prediction program multi-coil [55] to infer coiled coil regions, which are classified as SDs in the modified system. The system also identifies fibrous structures, collagen-like or α-keratin-like sequences, and classifies them as KDs. For this purpose, the query protein is checked whether it contains Pfam domains [42] corresponding to collagen (PF01391) or α-keratin (PF00038). If the query is a Swiss-Prot entry, the trans-membrane regions, signal peptides, and transit peptides are identified from the annotations and are regarded as KDs.

The modified DICHOT system was applied to all 20,333 human proteins in the Swiss-Prot database (version 56.6). The other model organisms used together with the numbers of proteins are: *Drosophila melanogaster *[56], 21,030; *Caenorhabditis elegans *[57], 23,662; *Arabidopsis thaliana *[58], 30,690; *Oryza sativa *[59], 26,887; *Schizosaccharomyces pombe *[60], 5,289; *Saccharomyces cerevisiae *[61], 5,880; *Escherichia coli *[62], 4,133; *Bacillus subtilis *[63], 4,104; *Pyrococcus furiosus *[64], 2,065.

The Swiss-Prot database was consulted to obtain information on subcellular localizations from the "SUBCELLULAR LOCATION" lines in the comment (CC) section, as well as that on AS from the variable sequence ("VAR_SEQ") lines in the feature table (FT) section. Both ends of an AS-associated variable sequence were examined if they fall in ID regions or SDs. While AS positions were taken from Swiss-Prot, the positions of exon boundaries were acquired from the Ensembl database, because exon boundaries except for those involved in AS are not available in Swiss-Prot. The locations of the other events in Figure 5 were also taken from the FT section of Swiss-Prot.

Using the Pfam database version 23.0 [42], HMM searches of Pfam domains were conducted against query sequences and the residue-wise fractions of Pfam coverage in the KDs, CDs, and ID regions of human proteins were estimated from the results.

## Authors' contributions

SF developed the DICHOT system, applied it to several model organisms, and also drafted the manuscript. KH participated in drafting the manuscript and carried out the assessments of the results. KH conducted the analysis on subcellular localizations. TG supervised the work. KN participated in the coordination of the study and the assessments of the results, and drafted the manuscript.

## Authors' information

The corresponding author, Satoshi Fukuchis' current address is: Department of Bioinformatics, Maebashi Institute of Technology, Kamisadori 460-1, Maebashi, Gunma 371-0816, Japan and his email address is sfukuchi@maebashi-it.ac.jp.

## Supplementary Material

Additional file 1 **Figure S1**. Fractions of IDPs with contiguous ID regions longer than the specific length. Click here for file

Additional file 2 **Table S1**. The number of human proteins in each category subcellular localization. Click here for file

Additional file 3 **Figure S2**. Fractions of IDPs with contiguous ID regions longer than the specified length in different subcellular localizations. Click here for file

Additional file 4 **Figure S3**. Fractions of AS and exon boundaries occurring in ID regions. Click here for file
