# Supplementary material for: Binary classification of protein molecules into intrinsically disordered and ordered segments
Source: BMC Struct Biol. 2011 Jun 22;11:29. doi: 10.1186/1472-6807-11-29 (PMC3199747; doi:10.1186/1472-6807-11-29)
Supplement: Additional file 1 — Figure S1. Fractions of IDPs with contiguous ID regions longer than the specific length. [file 1472-6807-11-29-S1.PDF]

Fig. S1

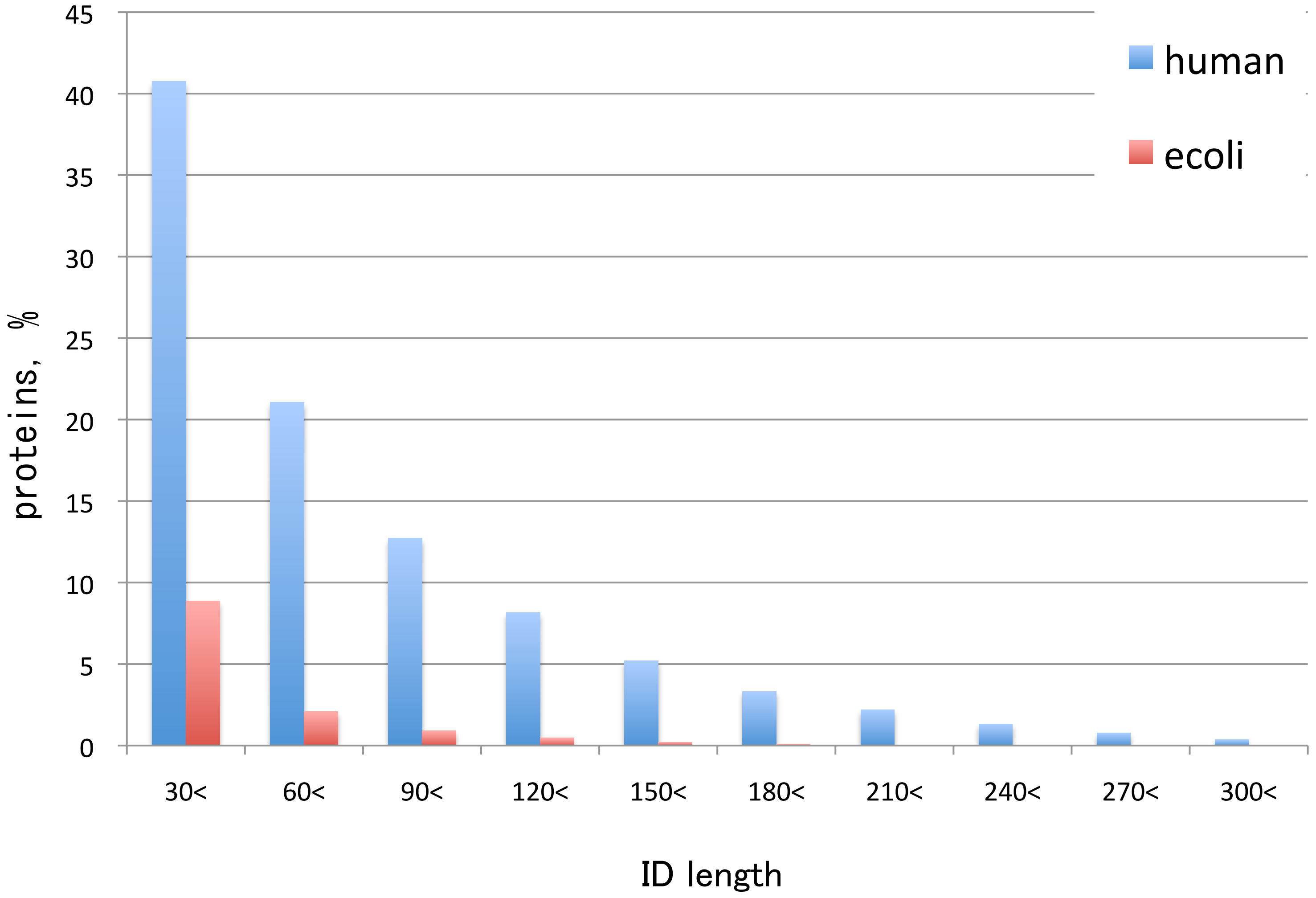

Figure S1. fractions of IDPs with contiguous ID regions longer than the specific length. The abscissa indicates ID regions longer than specific number of residues. The length distributions of ID regions as in Figure 3, but human and E. coli proteins longer than 200 and shorter than 400 residues, are only used.
