# Supplementary material for: Binary classification of protein molecules into intrinsically disordered and ordered segments
Source: BMC Struct Biol. 2011 Jun 22;11:29. doi: 10.1186/1472-6807-11-29 (PMC3199747; doi:10.1186/1472-6807-11-29)
Supplement: Additional file 2 — Table S1. The number of human proteins in each category subcellular localization. [file 1472-6807-11-29-S2.PDF]

Table S1. The number of human proteins in each category subcellular localization.

Localization	#protein
Nucleus	3016
Nucleus&Cytoplasm	778
Cytoplasm	1052
Plasma membrane	4243
Secreted	1398
ER/Golgi	116
Mitochondrial membrane	180
Mitochondria	408
