# Supplementary material for: Binary classification of protein molecules into intrinsically disordered and ordered segments
Source: BMC Struct Biol. 2011 Jun 22;11:29. doi: 10.1186/1472-6807-11-29 (PMC3199747; doi:10.1186/1472-6807-11-29)
Supplement: Additional file 3 — Figure S2. Fractions of IDPs with contiguous ID regions longer than the specified length in different subcellular localizations. [file 1472-6807-11-29-S3.PDF]

Fig. S2

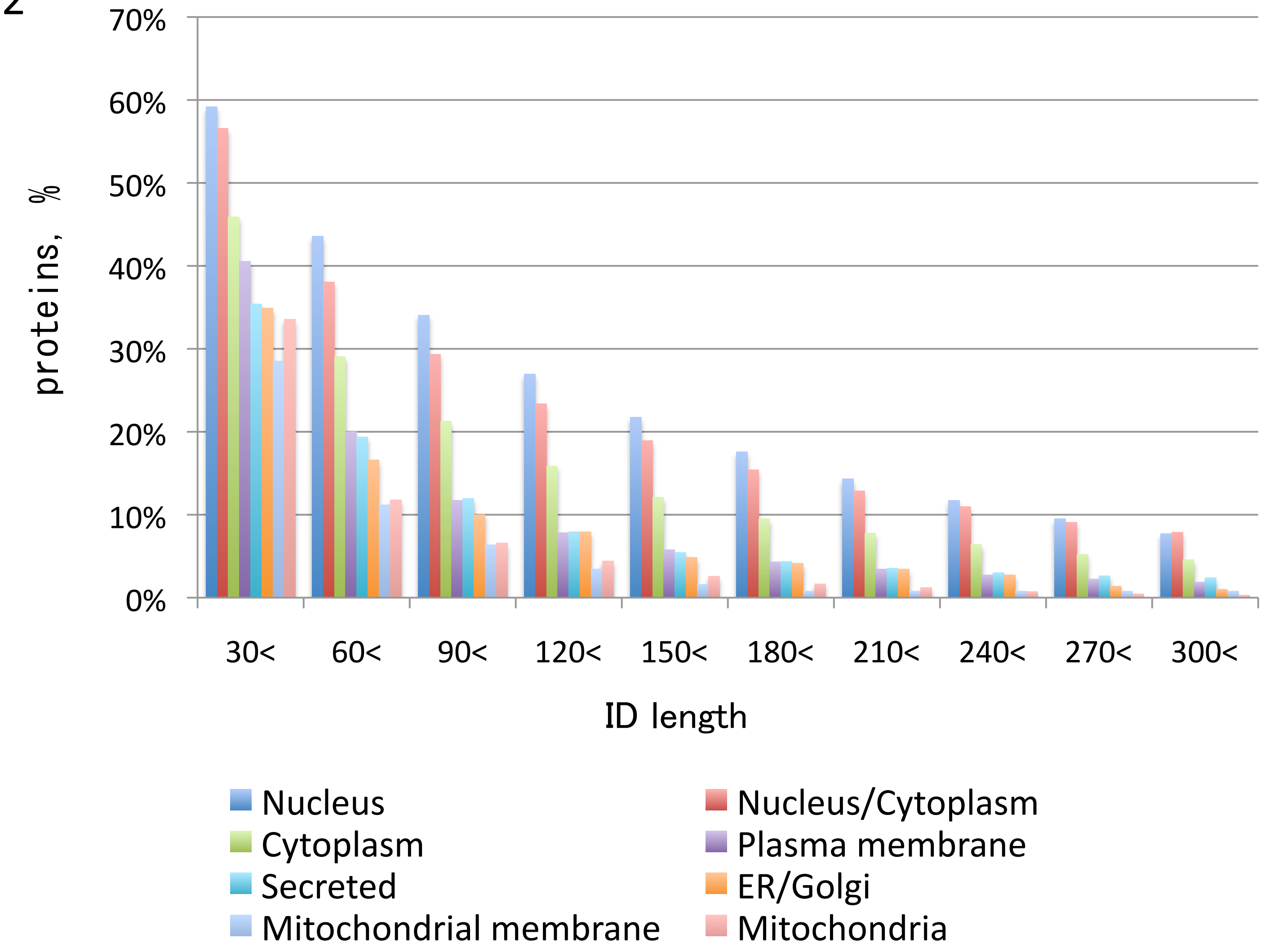

Figure S2. Fractions of IDPs with contiguous ID regions longer than the specified length in different subcellular localizations. The length distribution of ID regions as in Figure 3, but human proteins in different subcellular localizations, is presented. The abscissa indicates ID regions longer than the specified number of residues.
