# Supplementary material for: Binary classification of protein molecules into intrinsically disordered and ordered segments
Source: BMC Struct Biol. 2011 Jun 22;11:29. doi: 10.1186/1472-6807-11-29 (PMC3199747; doi:10.1186/1472-6807-11-29)
Supplement: Additional file 4 — Figure S3. Fractions of AS and exon boundaries occurring in ID regions. [file 1472-6807-11-29-S4.PDF]

Fig. S3

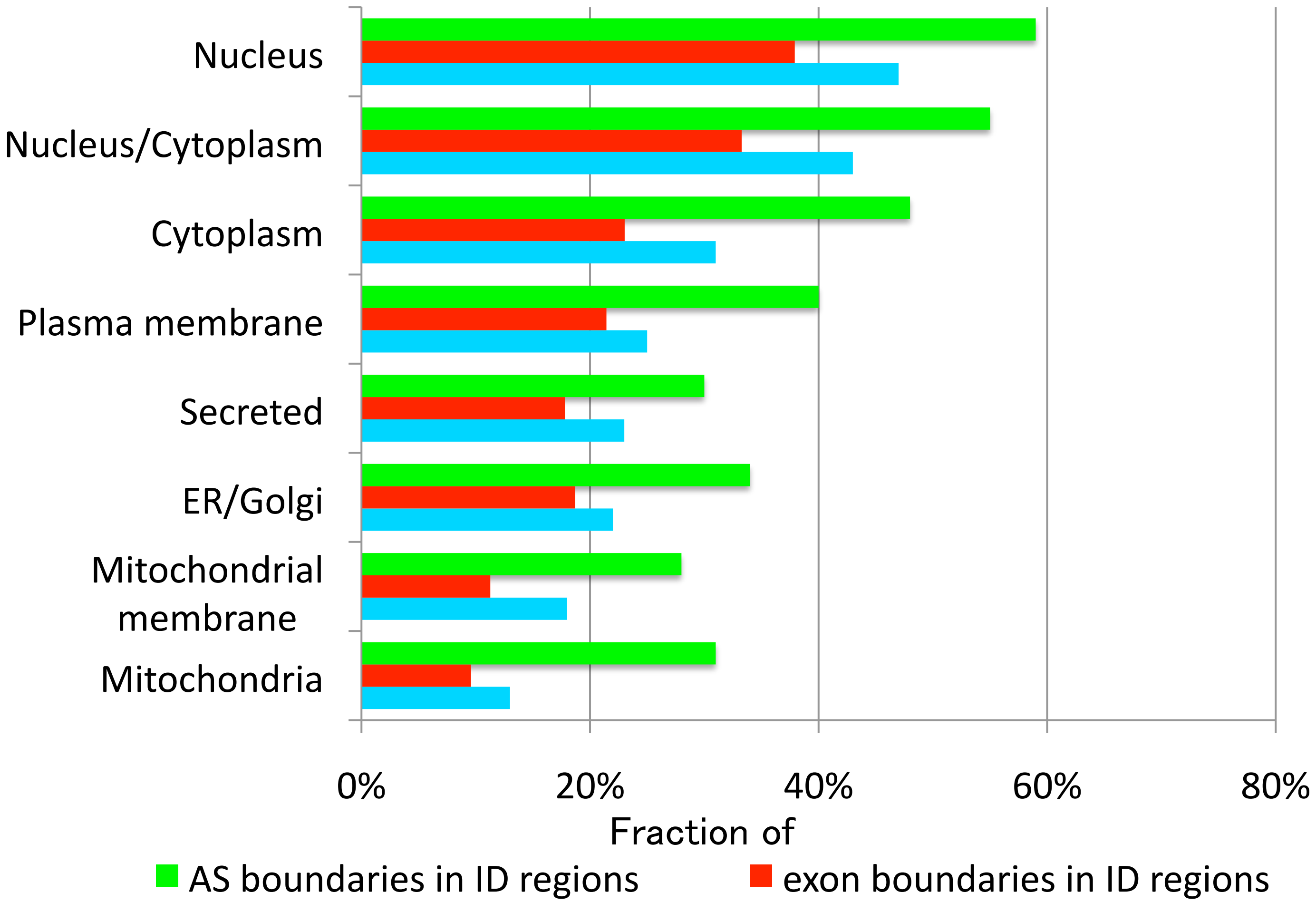

Figure S3. Fractions of AS and exon boundaries occurring in ID regions.

The green bar labeled Nucleus, for instance, indicates the fraction of the AS boundaries of nuclear proteins occurring in ID regions, instead of in SDs. Similarly, the red bar below indicates the fraction of the exon boundaries of nuclear proteins occurring in ID regions. If AS or exon boundaries of proteins in each subcellular localization were evenly distributed in SDs and ID regions, the fraction would be identical to that of ID regions (blue bar).
